# Python for gene expression

**DOI:** 10.12688/f1000research.53842.1

**Published:** 2021-08-31

**Authors:** Leonid Bystrykh

**Affiliations:** 1 ERIBA, University Medical Center Groningen, University of Groningen, Groningen, 9713 AV, The Netherlands

**Keywords:** differential gene expression, single cell expression, python, R, limma

## Abstract

Genome biology shows substantial progress in its analytical and computational part in the last decades. Differential gene expression is one of many computationally intense areas; it is largely developed under R programming language. Here we explain possible reasons for such dominance of R in gene expression data. Next, we discuss the prospects for Python to become competitive in this area of research in coming years. We indicate that Python can be used already in a field of a single cell differential gene expression. We pinpoint still missing parts in Python and possibilities for improvement.

## Introduction

Fundamental breakthrough in sequencing technologies in late 1990 promoted explosive growth of the data accumulated in biology in the last two decades. First, the introduction of expression microarrays has initiated accumulation of genome-wide gene expression data from different organisms, which stimulated creation of dedicated databases and development of computational tools for its analysis. Second, a more substantial wave of expression data arrived along with progress in high-throughput DNA sequencing, ^1(p), 2^ which demanded even bigger data storage and more sophisticated means of maintenance, programming support and analysis. ^3- 5^ This coincided with improved performance of our computers accompanied by the development of programming languages, especially those that paid attention to the biology-specific demands in data analysis, such as R and Python. ^6- 8^ Although the current list of known programming languages is approaching 400 (compiled by Wikipedia), there are only a handful of languages supporting dedicated biology-oriented packages ( Table 1). Thus, the theoretical choices for languages with specialized support of biological applications is still very limited.

**Table 1.** Programming languages supporting biological packages, their names and major focus.

Programming language	Name	Major applications
C++	Bio++	Sequencing and phylogenetics
Java	BioJava	DNA/RNA/Protein sequence analysis
JavaScript	BioJS	Mostly Sequence analysis, some elements of GO and visualizations
Perl	BioPerl	Mostly sequencing related
PHP	BioPHP	Mostly sequencing related
Python	BioPython Snakemake ^^1^^	Mostly sequencing related Special package to reproducibly organize complex pipelines
Ruby	BioRuby	Mostly sequencing related
R	Bioconductor	Huge collection of different kinds, no specific subject. Not really for sequencing

^1^ Snakemake ^9^ is python based workflow managing system, in other words pipelines organizing software, which is more than a regular package (compared to others mentioned in this table).

It is also worth mentioning Bioconda ^10^ installation package, which assists finding and installing various tools for biological data analysis. It is a sort of a spin-off the Anaconda installation package for Python, but with extended spectrum of options and possibilities.

## R and Perl

Python, as a fully functional and ready for tasks of general programming, arrived with as version 2.0 in 2000. By that time R was already a well-established language in bioinformatics, especially for statistical applications, see for instance. ^11^ At that time Perl was probably the most used programming language in genome biology (especially suited for string operations on DNA, RNA and protein sequences), due to its better computational performance, ^12^ and it stays strong in a field of genome sequence analysis even now, although it’s difficult syntax and accumulating problems with maintenance of the packages has caused a gradual decline in popularity (as for instance recorded in codementor.io site for the worst programming languages). Nevertheless, Perl scripts can be seen on the back pages of Ensembl BioMart and also Unigene pages.

Since the introduction of the Affymetrix expression microarrays in the 2000s, it immediately required means of programming development; and the R language with its strong statistical component was ready for the immediate use in the field of expression data analysis. The key elements in e establishing R as a standard language in the field was resolving a problem of (microarray) data normalization ^13^^,^^14^ and subsequent implementation as an R package (for instance Bioconductor preprocessCore ^15^). Publishing the Limma package ^16, 17^ was absolutely crucial for success of R in that area; it resolved a problem of a small sample size for microarray expression data systematically provided by biologists at that time. Since 2003-2005 clear separation of tasks became visible: Perl was focused on tasks of sequencing analysis, while R covered statistics and differential analysis, including expression microarrays.

## Limma package

Since the first publication of the Limma package by the group of Gordon Smith, ^16^^,^^18^ it became a central and indispensable element of major differential expression protocols in R for at least a decade since its introduction. In the early 2000s, microarrays were expensive and many labs could afford only a limited number of samples to analyze. The core issue resolved by this package was how to bypass a dilemma of a small number of samples in groups and still obtain credible and statistically validated results. Suppose you try to apply a t-test to a set of data with only 2 or 3 replicates per group and a total number of tests up to 20000 times. This is equivalent to analyzing expression microarray data containing 20000 gene expression in a series of 2 controls and 2 experimental samples. Regular t-test with correction for multiple testing has little chance for success. Limma has two essential steps circumventing this problem. One is using a linear model for a data fit for the entire table of data, followed by using empirical Bayesian statistics to recalculate probabilities based on the entire distribution of the expression data for all genes across the expression array.

This concept was directly inherited in later protocols for the bulk RNAseq analysis with edgeR package. ^19^ Namely, the Voom function in edgeR implements very similar steps of data conversion compared to the original Limma package. Another popular protocol in R, namely deseq2 ^20(p2)^ (as well as deseq) used a similar approach, although not directly copying the Limma algorithms. Details can be found in corresponding tutorials to the packages in Bioconductor.

No packages were designed in other programming languages. This lack of diversity of choices created a monopoly of R protocols for the “classic” gene-expression analysis based on microarray data or bulk-RNAseq with limited number of samples per group.

Technically Python allows to “wrap” or quote other programming languages within its own scripts. Python can currently “quote” some lines from JavaScript, especially when ipynb file format is used. For R language there is a special wrapper, rpy2, ^21^ which can incorporate parts of the R functions within Python. Potentially, there is a possibility to wrap R-functions from any R package into Python. However, there is not a genuine alternative in another language, and besides, it is not a popular approach in current publications, which could be recommended to biologists as a standard protocol. Note, that by standard protocol we imply a script suggested by package developers, which can be followed by the user with “average” skills in programming.

Consequently, for quite a while the Python language had no usable application for the differential gene expression analysis, especially in times when expression microarrays and bulk RNAseq data with small sample numbers dominated the literature. Sporadically, one can find some reports with peculiar options available in Python. For instance, a “geometrical approach” was suggested a while ago for finding differentially expressed genes, ^22^ for which the implementation in jupyter notebook is also available. ^23^ A similar “geometric approach” is discussed in another publication ^24^ (although the later analysis was performed in R). Inspection of some of those scripts ^22^^,^^23^ reveals that the “geometric” approach rehearses a fold change statistics rather than eBayes probability approach and thus is not recommended.

## Why Python?

Indeed, if R and Perl performed so well, each in its own niche, why do we need Python after all? In fact, with further evolution of biological sciences more biologists realized the necessity of some elementary data analysis by themselves. Whereas R is still strong and powerful for professional statisticians, it is also recognized as a difficult language to learn and to comprehend (see for instance introduction in Quick-R, https://www.statmethods.net/). The same in part is true for Perl. Python, on the contrary was originally designed to be more human-friendly, more transparent, and a clearer computer language compared to Perl and R. More details of languages in comparison can be found on the Python official site ( https://www.Python.org/doc/essays/comparisons/). This gradually became recognized by the broad community of interested people, including all kinds of scientists and non-scientists in Universities, secondary education and other businesses. This made Python the most popular computer language in recent years (according to https://pypl.github.io/PYPL.html for instance).

The second useful feature of Python is how functions are organized and stored. Unlike R, where each individual contributor writes their own package, and gradually it becomes a collection of millions of functions, often redundant. Python has a policy of bigger consortia and bigger collections of functions within libraries with less redundancy in its content (although small packages also exist). The core packages like SciPy and Numpy collect long lists of useful functions for elementary math and statistics. They are universally used as a source of scientific and numeric functions. On top of these, other more dedicated libraries are developed, like Scikit-learn (a.k.a. Sklearn) package for machine learning applications, Pandas for file and table management, Statsmodels for various kinds of a model fitting. Regarding visualisations, most core options are in Matplotlib library, beyond that more specific illustrations could be found in Seaborn, others in Bokeh and so on. Noteworthy, Pandas, Statsmodels and Seaborn are stylistically similar and resemble R-style to some degree in their exterior. Unfortunately, Sklearn package currently does not support Pandas data frame data structures, although it can be worked around via Numpy array conversions.

## What is missing in Python for expression microarrays analysis?

Saying that the entire Limma package is missing in Python is a bit vague statement. It is important to specify what is exactly missing, what part of it cannot be replaced by existing alternatives. Typically, a microarray protocol is built in steps, many of which are already available in Python. Expression microarray data are deposited in public databases, the most known is GEO site, which also has a built-in tool GEO2R with an R script attached ^25^; the script would begin with package enabling fetching the data from the site. Next, data are converted to the table. Values and their distribution are inspected by checking the histogram, boxplots, and maybe MDS plot. It enables us to find out whether data are already log2-transformed and normalized (high or low scale of intensities, also whether data look reasonably normally-distributed or not) or has to be log2 transformed and normalized (equalized) to one another. If required, we add a step of log2-transform (available as core function in R) and quantile normalization (available in Limma and preprocessCore). All those mentioned steps are also available in Python (see Table 2 for details). Next, we define groups, then the model for our *lmFit* function. This is a sort of *lm* function available in Python statsmodels and core R, but *lmFit* works for entire table of data and it collects the results for an entire table as well. It is accompanied by another *contrasts.fit* step which is more of the same for specified groups of data. Further we have a function *eBayes*, which recalculates statistics obtained from the fit steps above and finally generates Bayes corrected values for significance. This is essentially the heart of Limma, which is not available in Python in any form. At last, *topTable* function organizes a final table of differential expressions, what we well know from our own work and publications. Further, it can be decorated by more illustrations, like volcano plot or another PCA plot, etc. All those decorative functions can be done in Python as well. To summarize, the *lmFit* and *eBayes* are the only critical elements missing in Python precluding its use for microarray gene expression analysis.

**Table 2.** Steps and functions for differential expression microarrays analysis in R and analogues in Python.

Step	R package/function	Python analogue
Fetch data from GEO	GEOquery (Bioconductor)	GEOparse
Visualize data	hist(), boxplot()	plt.hist(), plt.boxplot() (Matplotlib)
Log2 transform	log2()	log2 (Math)
Quantile normalization	normalizeBetweenArrays() (Limma), normalize.quantiles() (preprocessCore)	Not directly available, the procedure is described in detail, it can be written as custom code
Model fit	lmFit(), contrasts.fit() (Limma)	Not directly available, may be made from statsmodels package functions.
Calculate significance	eBayes() (Limma)	Missing
Generate differential expression table	topTable() (Limma)	Missing, but can be written as custom script
Extra visualizations	Volcanoplot (Limma), PCA (multiple packages)	Basic plots in Matplotlib, plt.scatter(), PCA, MDS, in SciKit-learn

Note: packages for functions are in brackets behind the function.

## What is missing in Python for bulk RNAseq analysis?

Major packages in RNAseq differential gene expression analysis in R utilize the concepts/functionalities implemented in Limma package directly or indirectly. For instance, edgeR package designed for bulk RNAseq differential expression imports Limma as a dependent package and uses elements of it. The basic steps are slightly different, but the outline is very much the same. The first step is usually either trivial read file function or read raw mapped data as series of separate files, and makes a table out of it. The data can be either raw read counts, coming directly from the step of sequencing reads counts per transcript, or corrected by transcript length (in RNA seq it is essential for comparison expression levels across different genes). Unlike to microarray data, which are the smallest expression data among all others, RNAseq primary data are much bigger in size, and they contain lots of low-level or not expressing genes. Consequently, there is a step removing genes with low read values. Those genes are useless in terms of differential analysis and only overload the memory. Since different samples in RNAseq can have different read coverage, and also a different number of detected genes (above zero), the whole philosophy of normalization is rather complicated. However, the resulting procedure of normalization is reduced to familiar log2-transform step followed by dividing all gene-expression values by so-called normalizing factors. Fortunately, algorithms of finding normalizing factors are mostly well described, especially for deseq2 (an outline can be found in Maza, 2016 ^26^). Therefore, it is possible to write a custom script in any available language including Python, which would recapture this sort of the normalization step. When normalization is done, the next important step is estimation of data dispersion. This step is rather complicated in details not suitable for this type of article. In edgeR there are many alternative options for this step available. After that the step of statistical estimation of significance comes to a play. The resulting differential expression table follows the steps of a *topTable* from Limma. If we inspect options for Python, we will find out that similar to microarrays Python largely misses a step of dispersion analysis, estimation of fold change statistics, and statistical significance. Other steps can be replaced by known functions or custom scripts ( Table 3).

**Table 3.** Steps and functions for RNAseq DE analysis in edgeR and analogues in Python.

Step	R package/function	Python analogue
Read the data from file	read.csv(), read.table()	Read_csv (pandas)
Visualize data	hist(), boxplot()	plt.hist(), plt.boxplot() (Matplotlib)
Convert to special data format	DGElist()	Not used
Calculate normalizing factors (normalize and log-transform)	calcNormFactors()	not directly available, the procedure described for deseq2 can be written as custom code
Estimate dispersion	Many kinds of estimateDispersion()	Not available
Calculate significance	exactTest()	Not available in this context
Generate differential expression table	topTable() (Limma)	Missing, but can be written as custom script
Extra visualisations	Volcanoplot (Limma), PCA (multiple packages)	Basic plots in Matplotlib, plt.scatter(), PCA, MDS, in SciKit-learn

Note: deseq2 protocol makes steps from normalization to differential expression table in one function.

## Single cell RNAseq in R

Since R set a good trend for making all previous protocols for differential gene expression, it also pioneered a single cell gene expression protocol. Out of many protocols generated so far, the most frequently used are Scater, ^27^ Scran, ^28^ Seurat ^29^^,^^30^ and Monocle. Scater and Scran packages are built on a common data type, SingleCellExperiment, ^31^ and thus can be combined in one script using the same data type (which is often the case). In contrast, Seurat is built on its own data type and aims to be a self-sufficient package. It is currently a popular choice; it is especially appreciated for good tutorials and colorful illustrations, although integration of Seurat with other tools or packages is limited.

Single cell protocol for differential gene expression likely originated from bulk-RNAseq, but it diverged from its ancestor in subsequent years. Some steps in both protocols are still common, some are different. For instance, SC-RNAseq acquired a step to check sample quality and removal of bad quality samples (which are gene expressions per cell in this case). Normalization and log2-transform are carried out in a similar fashion as in bulk RNAseq, although normalization became even more simple: samples are usually adjusted to the median read counts across entire sets of data and proportional to the detected genes per sample. Next, there is tedious step of identifying groups of cells for differential expression analysis and other characterization. Unlike other differential expression protocols, SC-RNAseq is aimed on characterization of cells, not genes, and possible discovery and/or classification of existing cell types. This is a unique and specific chapter for SC-RNAseq only. The differential gene expression is performed using regular statistical tests (there is no particular preference to those). Close to the end of the SC-RNAseq protocols, we observe increasing diversity of options and specific interests.

It is important to emphasize that while R scripts in general often serve as standard protocols (or claimed to be a standard protocols), it is not really the case for bulk RNAseq and SC-RNAseq protocols. Currently used packages are known to differ substantially in detail, as well as the results of those data analysis. Therefore, we cannot pinpoint any particular protocol as standard in the field of differential gene expression analysis in R. This and availability of alternative commercial protocols for differential gene expression might be an extra source of the data irreproducibility problem in this particular field of research.

## Single cell RNAseq in Python

Unlike expression microarrays or a bulkRNAseq experiment, a single cell expression experiments contains lots of samples (and samples in each group if groups are defined). Therefore, the major constraint, which existed in early years, namely circumventing a dilemma of small sample numbers does not apply here. With hundreds of samples per group we can apply regular statistics, which is available in Python and other languages. Therefore, with the introduction and development of a single cell differential gene expression analysis it became possible to assemble the entire protocol from available Python functions. Surely, the development of a dedicated package might facilitate the use and popularity of Python for such analysis. In this regard, it is worth mentioning the release of the very first dedicated package of this sort, namely Scanpy. ^32^ Scanpy basically follows the sequence of data transformation and analysis from Seurat. They both provide tutorials on the same data sources, which makes them especially attractive for use and open for cross analysis and cross validation. Hopefully Scanpy will stimulate program developers for more interesting projects in a field of single cell analysis.

There is also an alternative to this, namely create specific functions, which can be recruited with regular tools and functions already available in different packages in Python. Table 4 shows a sketchy comparison of how minimal protocol is organized in Seurat, Scanpy and reassembled from scratch.

**Table 4.** Steps and functions for SC-RNAseq DE analysis in Scater, Scanpy and regular Python.

Step	Seurat	Scanpy	Python
Read the data from file	read.csv() *	scanpy.read_csv	pandas.read_csv ()
Convert to special data format	CreateSeuratObject()	Already converted as AnnData	Keep as pd. DataFrame
Filter off outliers	Regular R functions	FilterCells(), FilterGenes()	Use general pandas functions for subsetting by threshold values
Normalize and log-transform	NormalizeData()	normalize_total()	normalize from Sklearn or self-made script
Remove invariant genes	FindVariableFeatures()	highly_variable_genes()	Use pandas DataFrame filter by *var* value. Use VarianceThreshold() from Sklearn
Scale gene expressions to 0-1 interval	ScaleData()	scale()	Normalize() in Sklearn
Run PCA, estimate significant components	RunPCA(), JackStraw()	pca()	Sklearn PCA()
Find or use predefined clusters	FindNeighbors(), FindClusters()	Import leiden, other options possible	Different options in Sklearn.cluster
Run tSNE, visualize clusters	RunTSNE(), TSNEplot()	Prefers UMAP (as imported package)	tSNE and other options in sklearn.manifold
Perform differential expression check	FindMarkers(), FindAllMarkers()	Build in options for Wilcoxon, t-test, logistic regression	t-test, oneway ANOVA, Wilcoxon, Kruskal-Wallis etc. in scipy.stats, RandomForest, ADAboost in sklearn

* read.csv() in Seurat used for regular table read. Read10X() is for reading matrix data format.

Currently this field is wide open for more examples of Python-base analysis for differential expression in single cells. Some simple examples can be found on GitHub as *Extended data* (which should not be taken as a standard protocol for the differential expression). Researchers should not be confused by the fact that different protocols result in different lists of the differentially expressed genes. This is already described for different RNAseq protocols in R, caused for instance by differences in normalization ^26^^,^^33^ or other steps. ^34^ The differences between those protocols are acceptable since we use not identical, but only comparable, steps and functions. The major and most prominent differentially expressed genes are usually consistent and not prone to variation upon changing options within protocols or between those. In addition, the researcher can also try artificial data to check details of the protocols on reproducibility and consistency. ^35^

## Concluding remarks

Even though R remains the major language for differential gene expression analysis, further rise of Python popularity in biological applications is expected in the coming years. Regarding single cell expression data, Python has broad possibilities for data analysis. Moreover, the rise and diversification of the single cell protocols will require more programming flexibility, where Python might offer more options with respect to R. This is also dependent of community efforts within Python developers. We might expect some restructuring of existing packages and emergence of specialized dedicated packages in the direction of the single cell analysis. The time is right for more efforts in Python applications. Regarding flexibility, it is essential to keep all options open for integrating functions from different existing and future packages.

More active use of Python in biological studies will certainly improve transparency and reproducibility of currently used protocols for differential gene expression and beyond. It is also a satisfying feeling that biological science makes a substantial shift from descriptive empirical style into a more exact and analytical mode.

## Data availability

### Underlying data

No data is associated with this article.

### Extended data

Extra information and example scripts are available: https://github.com/LeonidBystrykh/PY4DE/tree/main.

Archived scripts as at time of publication: http://doi.org/10.5281/zenodo.5044809. ^36^

License: GPL-2
