# Supplementary material for: Python for gene expression
Source: F1000Res. 2022 Jun 23;10:870. Originally published 2021 Aug 31. [Version 2] doi: 10.12688/f1000research.53842.2 (PMC9130758; doi:10.12688/f1000research.53842.2)
Supplement: Peer review history

# Peer review: Reviewer response for version 2

**DOI:** 10.5256/f1000research.134735.r141893

**Published:** 2022-06-24

**Authors:** Sergio Peignier

**Affiliations:** 1 Université de Lyon, INSA Lyon, INRA, Villeurbanne, France

Dear Leonid Bystrykh,

 The paper is very well written, very clear, and will be an important contribution to the field. I have no more comments regarding the article,

 Kind regards,

 Sergio Peignier

Is the topic of the opinion article discussed accurately in the context of the current literature?

Yes

Are arguments sufficiently supported by evidence from the published literature?

Yes

Are all factual statements correct and adequately supported by citations?

Yes

Are the conclusions drawn balanced and justified on the basis of the presented arguments?

Yes

Reviewer Expertise:

Gene regulatory networks inference, gene expression analysis, hyperspectral image analysis, Subspace clustering

I confirm that I have read this submission and believe that I have an appropriate level of expertise to confirm that it is of an acceptable scientific standard.

# Peer review: Reviewer response for version 1

**DOI:** 10.5256/f1000research.57265.r136994

**Published:** 2022-05-24

**Authors:** Yasha Hasija

**Affiliations:** 1 Delhi Technological University, Delhi, India

The article "Python for gene expression" discusses the applicability of Python and R for gene expression data analysis. Beginning with a brief history of several programming languages and their compatibility with biological problems/data, the article then discusses their compatibility with biological problems/data. The authors then describe the advantages of R packages for the processing and statistical analysis of big expression data, as well as their replacement in Python. The article concludes that the Python programming language has wide use in biological data processing processes and that the scientific community should consider adopting it.

 The piece is well-written and effectively conveys its intended message. A few of my recommendations are:

- The sections on microarray data, RNAseq data, and SC-RNAseq data analysis describe the application of R packages and the limitations of Python due to the absence of a few libraries. It would be interesting to list a few advantages of using Python for bulk data processing.
- Also, advantages of Python over R in terms of automation, integration, and application development can be included.

Is the topic of the opinion article discussed accurately in the context of the current literature?

Yes

Are arguments sufficiently supported by evidence from the published literature?

Yes

Are all factual statements correct and adequately supported by citations?

Yes

Are the conclusions drawn balanced and justified on the basis of the presented arguments?

Yes

Reviewer Expertise:

Bioinformatics, Machine Learning, Polymorphisms

I confirm that I have read this submission and believe that I have an appropriate level of expertise to confirm that it is of an acceptable scientific standard.

# Peer review: Reviewer response for version 1

**DOI:** 10.5256/f1000research.57265.r136995

**Published:** 2022-05-17

**Authors:** Sergio Peignier

**Affiliations:** 1 Université de Lyon, INSA Lyon, INRA, Villeurbanne, France

Dear Leonid Bystrykh,

 The opinion article "Python for gene expression" is well written, and clear, it provides an interesting historical and contextual description and explanation for the dominance of R in differential gene expression analysis, and it also clearly points the interest and benefits of developing python projects dedicated to differential gene expression analysis.

 I hope the following remarks will be useful to improve this interesting paper.

 Kind regards,

 Sergio Peignier

- Maybe you can show from the title that the paper is mostly oriented towards differential gene expression analysis (e.g., "Python for differential gene expression").
- In Table 1 you can replace the column "Name" by "Main library" or something like this to be more explicit.
- "such as R and Python. ^6- 8^" <- maybe keeping citations for R and Python separated will give a better insight to the reader.
- "approaching 400 (compiled by Wikipedia)" <- consider adding a citation.
- "decline in popularity (as for instance recorded in codementor.io site for the worst programming languages)" <- consider adding a citation.
- "in e establishing" <- "in establishing".
- "One is using a linear model [...] the expression array." <- consider adding a citation to the paper.
- "Potentially, there is a possibility to wrap R-functions from any R package into Python." <- there are some DEseq2 wrapped versions available e.g., GReNaDIne: Data-Driven Approaches to Infer Gene Regulatory Networks in Python ( gitlab link).
- "peculiar options available in Python" <- Maybe  "specific" instead of " "peculiar"?
- "rehearses a fold change statistics " <- "rehearses fold change statistics".
- The following sentence could be clarified and a justification or citation to support it could be incorporated: "approach rehearses a fold change statistics rather than eBayes probability approach and thus is not recommended."
- Regarding the comparison between Python and other languages,
  "This essay was written sometime in 1997. It shows its age. It is retained here merely as a historical artifact. ( https://www.Python.org/doc/essays/comparisons/)"  the website that was cited by the author states: " **Disclaimer:** This essay was written sometime in 1997. It shows its age. It is retained here merely as a historical artifact.", so a more recent citation could be included instead. Moreover, I think that the comparison between python and R could be extended, in order to better support the idea that developing such a research field in Python would be valuable.
- Include citations for SciPy, Numpy, Scikit-learn, Pandas, statmodels, Matplotlib, bokeh and seaborn.
- "does not support Pandas" <- I would replace by "does not fully support Pandas" since some operations can be executed on pandas DataFrames, but the output is always a numpy array. 
- There are also classical methods for RNAseq normalization such as TPM, RPKM, that are not mentioned in the article, what is the place of such techniques in this context?.
- "Unlike other differential expression protocols, SC-RNAseq is aimed on characterization of cells, not genes, and possible discovery and/or classification of existing cell types" <- these datasets can also be used to study genes, and specially to infer Gene Regulatory Networks ^1^.
- SC-RNAseq also incurs in a missing values problem, that should be addressed by some pre-processing techniques, it could be interesting to discuss this problem. 
- Maybe you can try to include a few citations to new python programs dedicated to the analysis of gene expression, to support the idea that there is a community in computational biology and bioinformatics that is working in python. 
- The test scripts that are associated to the paper could be transformed into small tutorials, and could be very beneficial for the community.

Is the topic of the opinion article discussed accurately in the context of the current literature?

Yes

Are arguments sufficiently supported by evidence from the published literature?

Yes

Are all factual statements correct and adequately supported by citations?

Yes

Are the conclusions drawn balanced and justified on the basis of the presented arguments?

Yes

Reviewer Expertise:

Gene regulatory networks inference, gene expression analysis, hyperspectral image analysis, Subspace clustering

I confirm that I have read this submission and believe that I have an appropriate level of expertise to confirm that it is of an acceptable scientific standard.

## References

[1] : A scalable SCENIC workflow for single-cell gene regulatory network analysis. *Nat Protoc* .15(7) : 10.1038/s41596-020-0336-2 2247-2276 10.1038/s41596-020-0336-2 32561888
